# Non-Coding RNAs in the Etiology and Control of Major and Neglected Human Tropical Diseases

**DOI:** 10.3389/fimmu.2021.703936

**Published:** 2021-10-19

**Authors:** Ousman Tamgue, Cybelle Fodieu Mezajou, Natacha Njike Ngongang, Charleine Kameni, Jubilate Afuoti Ngum, Ulrich Stephane Fotso Simo, Fabrice Junior Tatang, Mazarin Akami, Annie Ngane Ngono

**Affiliations:** Department of Biochemistry, Faculty of Sciences, University of Douala, Douala, Cameroon

**Keywords:** non-coding RNAs, tuberculosis, HIV/AIDS, malaria, leishmaniasis, African trypanosomiasis, leprosy

## Abstract

Non-coding RNAs (ncRNAs) including microRNAs (miRs) and long non-coding RNAs (lncRNAs) have emerged as key regulators of gene expression in immune cells development and function. Their expression is altered in different physiological and disease conditions, hence making them attractive targets for the understanding of disease etiology and the development of adjunctive control strategies, especially within the current context of mitigated success of control measures deployed to eradicate these diseases. In this review, we summarize our current understanding of the role of ncRNAs in the etiology and control of major human tropical diseases including tuberculosis, HIV/AIDS and malaria, as well as neglected tropical diseases including leishmaniasis, African trypanosomiasis and leprosy. We highlight that several ncRNAs are involved at different stages of development of these diseases, for example miR-26-5p, miR-132-3p, miR-155-5p, miR-29-3p, miR-21-5p, miR-27b-3p, miR-99b-5p, miR-125-5p, miR-146a-5p, miR-223-3p, miR-20b-5p, miR-142-3p, miR-27a-5p, miR-144-5p, miR-889-5p and miR-582-5p in tuberculosis; miR-873, MALAT1, HEAL, LINC01426, LINC00173, NEAT1, NRON, GAS5 and lincRNA-p21 in HIV/AIDS; miR-451a, miR-let-7b and miR-106b in malaria; miR-210, miR-30A-5P, miR-294, miR-721 and lncRNA 7SL RNA in leishmaniasis; and miR-21, miR-181a, miR-146a in leprosy. We further report that several ncRNAs were investigated as diseases biomarkers and a number of them showed good potential for disease diagnosis, including miR-769-5p, miR-320a, miR-22-3p, miR-423-5p, miR-17-5p, miR-20b-5p and lncRNA LOC152742 in tuberculosis; miR-146b-5p, miR-223, miR-150, miR-16, miR-191 and lncRNA NEAT1 in HIV/AIDS; miR-451 and miR-16 in malaria; miR-361-3p, miR-193b, miR-671, lncRNA 7SL in leishmaniasis; miR-101, miR-196b, miR-27b and miR-29c in leprosy. Furthermore, some ncRNAs have emerged as potential therapeutic targets, some of which include lncRNAs NEAT1, NEAT2 and lnr6RNA, 152742 in tuberculosis; MALAT1, HEAL, SAF, lincRNA-p21, NEAT1, GAS5, NRON, LINC00173 in HIV/AIDS; miRNA-146a in malaria. Finally, miR-135 and miR-126 were proposed as potential targets for the development of therapeutic vaccine against leishmaniasis. We also identify and discuss knowledge gaps that warrant for increased research work. These include investigation of the role of ncRNAs in the etiology of African trypanosomiasis and the assessment of the diagnostic potential of ncRNAs for malaria, and African trypanosomiasis. The potential targeting of ncRNAs for adjunctive therapy against tuberculosis, leishmaniasis, African trypanosomiasis and leprosy, as well as their targeting in vaccine development against tuberculosis, HIV/AIDS, malaria, African trypanosomiasis and leprosy are also new avenues to explore.

## Introduction

Non-translated or non-coding RNAs (ncRNAs) are the transcripts of the genome that are not meant to be translated into proteins (1). They represent about 98% of total RNAs content within the human cells (2, 3) and were initially thought to be byproducts of transcription, therefore referred to as “Junk RNAs”. However, a growing body of evidence have unveiled the role of certain ncRNAs including microRNAs (miRNAs) and long non-coding RNAs (lncRNAs) as key regulators of gene expression, that is they can alter genes expression in a reversible, transmissible, and adaptative way, without modifying the DNA sequence (4). MicroRNAs are an abundant class of highly conserved small (18-25 nucleotides long) RNA species that generally downregulate the expression of their target genes at the post-transcriptional level. Mechanically, miRNAs bind in a sequence-specific manner to complementary regions in the 3’ untranslated region (3’UTR) of their target mRNAs, thereby triggering mRNA degradation or translation inhibition. In this way, a single miRNA can control the expression of several genes and a single gene expression can be controlled by several different miRNAs (5). Contrary to miRNAs, lncRNAs (at least 200 nucleotides long) are less studied, display poor sequence conservation and regulate the expression of their nearby proximal genes (Cis regulation) as well as distant genes (Trans regulation) at the chromatin, transcription and translation levels (6–8).

Several miRNAs and lncRNAs are emerging as key regulators of immune cells differentiation, activation, and function, including macrophages, dendritic cells and T lymphocytes (6). Some have been associated with specific disease conditions such as Cancer, cardiovascular, developmental (1, 9–12),neurodegenerative (13) and major infectious diseases such as tuberculosis and HIV/AIDS (14–16). There are however few or no studies addressing the role of ncRNAs in the etiology, diagnosis, treatment, or vaccine development for neglected human tropical diseases (NTDs) which are a group of less investigated infectious diseases especially common in tropical areas such as Africa and Southeast Asia where people do not have proper access to clean water and adequate means to discard their waste. In this review, we summarize most recent findings on the role of miRNAs and lncRNAs on major human tropical diseases including tuberculosis, HIV/AIDS and malaria. We also provide a first-time summary of our current understanding of the role of these ncRNAs in the etiology and control of neglected tropical diseases including leishmaniasis, African trypanosomiasis and leprosy. We also identify and discuss knowledge gaps that warrant for increased research effort.

## Tuberculosis

Tuberculosis (TB) is an infectious disease caused by *Mycobacterium tuberculosis* (*Mtb*) which has topped HIV as the deadliest infectious agent worldwide since 2017. Developing countries are highly burdened by this disease and further threatened by the emergence of multi-drug resistant and extensively drug resistant *Mtb* strains. Accurate diagnosis and effective treatment are the key elements to interrupt TB transmission (17). Several studies have linked miRNAs and lncRNAs to the onset and progression of TB, and some of those ncRNAs were identified as biomarkers for TB diagnosis or treatment (**Table 1**).

**Table 1:** Non-coding RNAs in the etiology and control of tuberculosis.

Role in tuberculosis	Non-coding RNA	Action	Reference
Etiology	miR-26-5p	Inhibition of innate immunity	(18)
	miR-132-3p		(18)
	miR-155-5p		(18, 19)
	miR-29-3p		(18)
	miR-21-5p	Suppression of inflammation	(18)
	miR-27b-3p		(18)
	miR-99b-5p		(18)
	miR-125-5p		(18)
	miR-146a-5p		(18)
	miR-223-3p		(18)
	let-7f		(18)
	miR-20b-5p		(18)
	miR-142-3p		(18)
	miR-33 locus	Inhibition of phagosome maturation and autophagy	(18)
	miR-27a-5p		(18, 20)
	miR-144-5p		(18)
	miR-889-5p		(18)
	miR-155-5p	Apoptosis inhibition	(18)
	miR--582-5p		(18)
Diagnosis	miR-769-5p	Downregulation in TB patients	(8)
	miR-320a		
	miR-22-3p		
	miR-423-5p	Upregulation in TB patients	(21)
	miR-17-5p		
	miR-20b-5p		
	lncRNA LOC152742		(22)
Therapeutic targets	lncRNAs NEAT1	Downregulation during drug treatment, association with disease improvement	(23)
	lncRNAs NEAT2		(23)
	lnrRNA 152742		(22)
	lncRNAENST00000429730.1	Downregulation during drug treatment, associated with complete inactivation of tuberculosis lesions from sputum negative patients	(24)
	lncRNA MSTRG.93125.4		(24)

### Non-Coding RNAs in the Etiology of Tuberculosis

**Figure 1:**
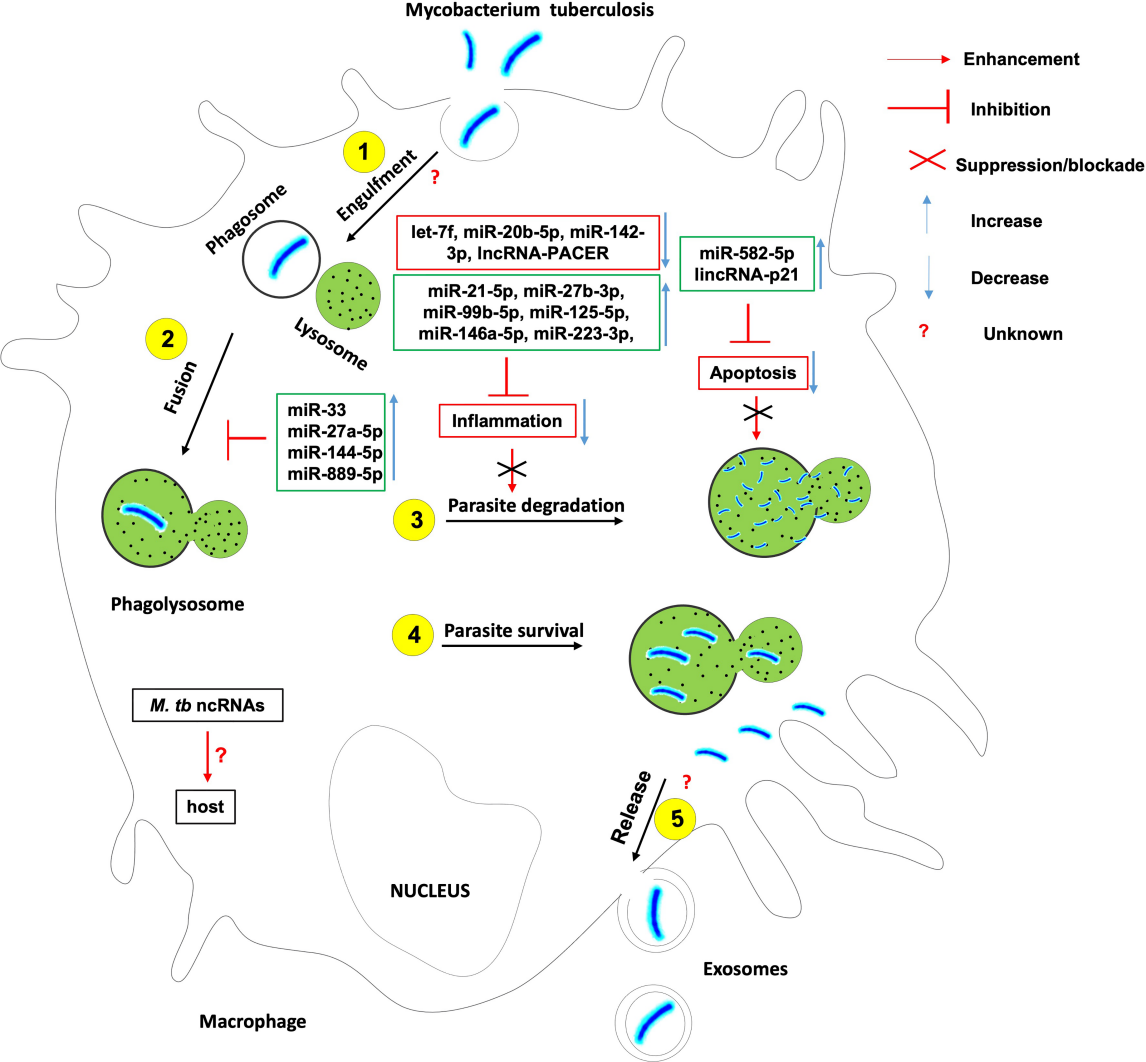
The role of non-coding RNAs in the etiology of tuberculosis. Several ncRNAs enhance host effector killing functions against mycobacterium tuberculosis and favor the bacterium survival and persistence within the infected host. There is knowledge gap about 1- host ncRNAs that regulate the bacterium engulfment within phagosomes and release in extracellular vesicles such as exosomes; 2- the role of Mtb-derived ncRNAs on the outcome of Mtb-macrophage interaction.

To fight a bacterial infection, host innate and/or adaptive immunity has to be activated. *Mycobacterium tuberculosis* like many other successful pathogens has evolved mechanisms to avoid the host immune system and ensure its intracellular survival and persistence. This is possible through the subversion of key ncRNAs that control the cellular and humoral processes enacted in host innate and adaptive immune response against *Mtb* (**Figure 1**).

The induction of a robust yet controlled inflammatory response plays a key role in the containment and eradication of the infection at an early stage. It was found that *Mtb* suppresses *inflammation via* the upregulation of miR-21-5p, miR-27b-3p, miR-99b-5p, miR-125-5p, miR-146a-5p, miR-223-3p, and the downregulation of let-7f, miR-20b-5p and miR-142-3p (18). *Mtb* HN878 infection of monocyte-derived macrophages (MDMs) induces the expression of the pro-inflammatory lncRNA-PACER (also known as lncRNA-Cox-2) which is a positive regulator of its proximal pro-inflammatory gene Ptgs-*2* (Also known as Cox-2) (25).

Macrophage and other phagocytes are the first immune cells to encounter *Mtb* in the lungs and make use of their phagocytic activity to engulf and destroy the invading *Mtb* using different cell death mechanisms. *Mtb* has developed several strategies to avoid killing within phagocytes. *Mtb* inhibits the phagosome maturation and autophagy *via* upregulation of miR-33 locus (18, 26), miR-27a-5p (18, 20), miR-144-5p and miR-889-5p. *Mtb* also evades the host defense by Inhibiting macrophage apoptosis (27) *via* the upregulation of miR-582-5p (28). It was also observed that *Mtb* HN878 infection of MDMs induces the expression of lincRNA-p21, a positive regulator of p53-dependent cell cycle arrest and apoptosis in numerous cell types (25, 29–32).

Much research is warranted to understand the contribution of lncRNA-PACER and lincRNA-p21 to the onset and progression of TB. Also there are growing body of evidence suggesting that *Mtb*-derived ncRNAs may be delivered to the host immune cells and affect their function. The contribution of such mechanisms to host immune evasion needs in-depth investigation.

### Non-Coding RNAs in the Diagnosis of Tuberculosis

Several miRNAs are decreased in the plasma of *Mycobacterium tuberculosis* infected patients compared to healthy individuals and are described as biomarkers for the diagnosis of tuberculosis (33). Amongst those miRNAs, miR-769-5p, miR-320a and miR-22-3p subsequently showed higher specificity (4, 33) at 90% sensitivity (92%), AUC (95%) and lower heterogeneity (I= 0%) in ethological-confirmation validation sets (17). Also, miR-423-5p, miR-17-5p, and miR-20b-5p were reported to be significantly increased in the serum of patients with tuberculosis and had the potential to be used to diagnose TB with an accuracy of 78.18% (21). The level of long noncoding RNA LOC152742 was found to be high in sputum and plasma of infected patients, hence could serve as novel biomarker for the diagnosis of active tuberculosis (22).

Sputum-negative pulmonary tuberculosis cases showing no clinical or microbial evidence contribute to the development and spread of active tuberculosis (34). Accurate diagnosis of sputum smear-negative cases of pulmonary TB remains very challenging. It was found that lncRNAs ENST00000429730.1 and MSTRG.93125.4 were upregulated in lung tissue samples collected from patients with sputum-negative pulmonary TB with high metabolic activity as compared to low metabolic activity according to FDG-PET/CT(Positron emission tomography with computed tomography (PET/CT) using fluorine-18-fluoro-deoxyglucose (FDG)) classification. Hence these lncRNAs might be potential biological indicators of metabolic activity in tuberculosis lesions for sputum-negative tuberculosis (24).

The emergence of multidrug-resistant strains of *Mtb* has further complicated the control and eradication of this disease. It was found that the plasma levels of miR-320a were decreased in drug-resistant TB patients as compared to pan-susceptible TB patients (33). Therefore, this miR-320a may serve as a biomarker for drug-resistant TB. Also, lncRNAs CTD-2331D11.3 and AC079779.5 were found to be increased in the Peripheral Blood Monocytic Cells (PBMCs) from patients infected with Multi-drug resistant *Mtb* strains (MDR-TB) when compared to patients infected with drug-sensitive strains, indicating this lncRNAs may be potential biomarkers for multi-drug resistant TB (35).

### Non-Coding RNAs as Therapeutic Biomarkers of Tuberculosis

The role of ncRNAs as potential host-directed therapeutical targets has been reviewed before (36). As a complement to that review article, recent studies have reported an increase in the expression of lncRNAs NEAT1 and NEAT2 in macrophages during *Mtb* infection. Their expression level was decreased during drug treatment, which was associated with improvement of the disease (23). The same observation was made with lnr6RNA 152742 which was upregulated in the plasma and sputum of patients and gradually downregulated in the course of the treatment (22).

Successful treatment of pulmonary tuberculosis is generally declared after absence of *Mtb* in sputum smear under microscopy and under culture. However, pulmonary TB lesions may still be harboring persisting slow growing, metabolically active but non-culturable bacilli that are less sensitive to chemotherapy agent and may cause TB relapse (37, 38) lncRNAs ENST00000429730.1 and MSTRG.93125.4 described as indicators of metabolic activity in tuberculosis lesions for sputum-negative tuberculosis (24), hence are potential biomarkers of complete inactivation of tuberculosis lesions, thus of complete cure of tuberculosis (24).

## Malaria

Malaria is a mosquito-borne disease caused by parasites of the genus *Plasmodium*. It is transmitted through the bites of infected female *Anopheles* mosquitoes. Five parasite species cause malaria in humans: *P. knowlesi*, *P.malariae*, *P.ovale*, *P. vivax* and P. *falciparum^1^*. The two last pose the greatest threat^2^. In 2019, around 229 million cases of malaria were recorded in the world with approximately 409 000 deaths^3^. Symptoms of malaria comprise fever, shaking chills, headache, muscle aches, and tiredness. Nausea, vomiting, and diarrhea may also be involved^4^. Due to the non-specificity of its symptoms, it is difficult to distinguish malaria from other acute febrile illnesses. Non-coding RNAs, which are specific, can be of great help in resolving this problem.

### Non-Coding RNAs in the Etiology of Malaria

**Figure 2:**
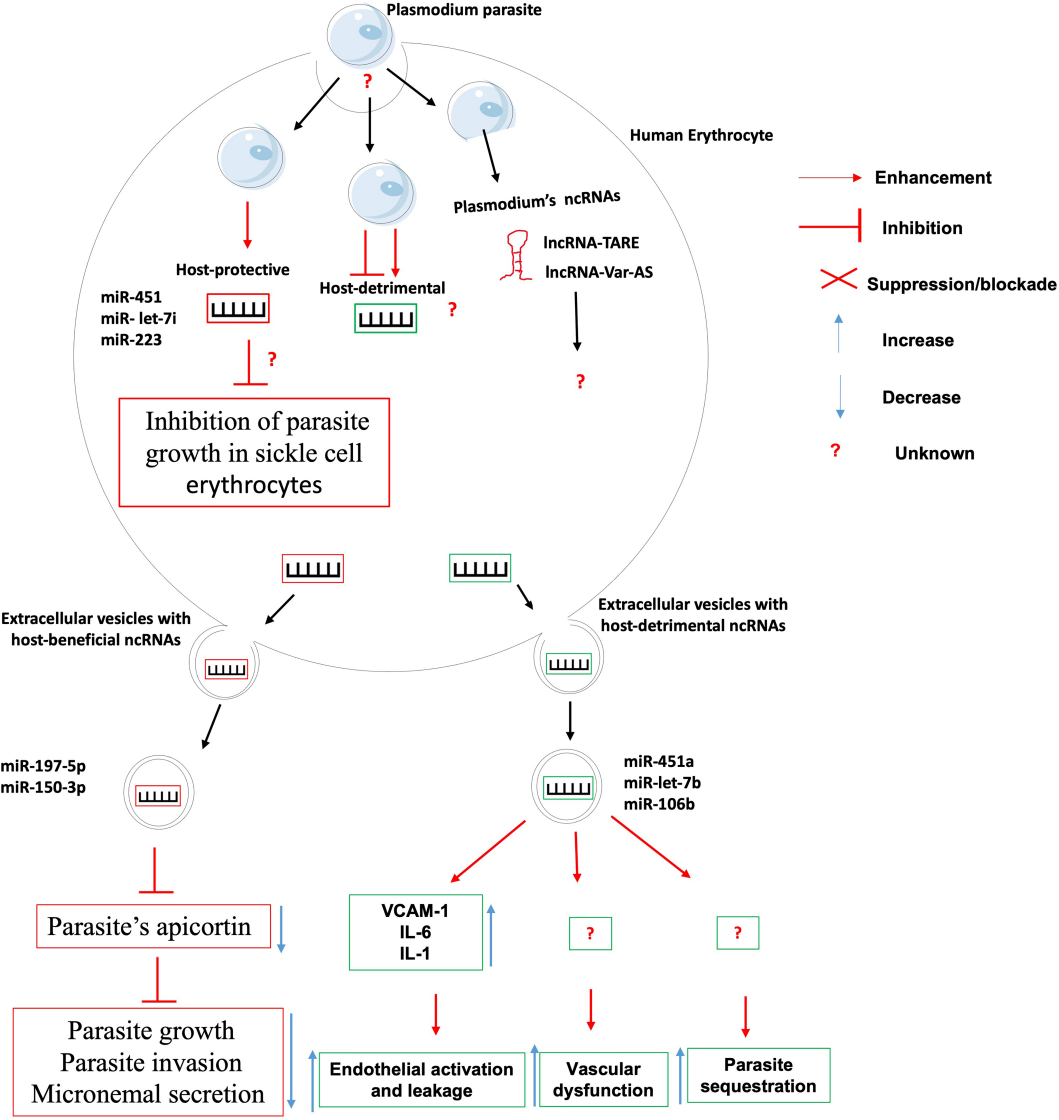
The role of non-coding RNAs in the etiology of malaria. Very few ncRNAs known to involve in the etiology of Malaria. There is knowledge gap about 1- host ncRNAs that regulate the uptake/entry of plasmodium within erythrocytes; 2- host-detrimental ncRNAs and their mechanisms of action; and 3- the role of plasmodium-derived ncRNAs on the outcome of plasmodium-erythrocyte interaction.

Despite the growing recognition of the contribution of ncRNAs in the etiology of infectious diseases, only a handful studies have specifically associated ncRNAs with the onset or progression of any clinical form of malaria, be it uncomplicated or cerebral (**Figure 2**).

The clinical outcome of persons infected with Plasmodium falciparum parasites depends on many factors including parasite sequestration in tissues, host systemic inflammatory responses, and vascular dysfunction. It was found that *Plasmodium falciparum*-infected red blood cells release extracellular vesicles (EV) loaded with functional host miR-451a, miR-let-7b and miR-106b. These miRNAs-loaded EVs are internalized by endothelial cells within which they induce the production of surface receptor vascular cell adhesion protein 1 (VCAM-1) and proinflammatory cytokines such as interleukin-6 (IL-6) and interleukin-1 (IL-1). This will lead to the promotion of endothelial activation, leakage and parasite sequestration as well as vascular dysfunction and pathology during malaria infection (39). Contrary to the abovementioned microRNAs that promote malaria pathology, it was found that erythrocytes-derived miR-197-5p and miR-150-3p reduced the parasite growth, invasion and micronemal secretion *via* a mechanism involving the inhibition of the expression of apicortin, a *Plasmodium falciparum’s* protein with putative microtubule-stabilizing properties (40). In addition, the resistance of sickle cell erythrocytes (carrying the HbS hemoglobin allele variant in either the heterozygous or the homozygous form) to infection by Plasmodium falciparum was associated to the expression of miR-451, miR- let-7i and miR-223 which were translocated into the parasite during its intraerythrocytic life cycle and negatively regulated the parasite growth (41). The mRNA targets of those microRNAs were however not investigated, neither was their contribution to the onset and maintenance of epigenetic marks such as DNA methylation and histone post-translational modifications on the promoter of possible target genes.

It was shown that knocking-out/down miR-155 led to reduced endothelial activation, reduced microvascular leak and preservation of blood-brain-barrier, reduced disease severity and improved survival in an experimental mouse model of cerebral malaria and an engineered human endothelialized microvessel system (42). Similarly, It was found that miR-19a-3p, miR-19b-3p, miR-540-5p, miR-142-3p and miR-223-3p were significantly upregulated in the brain of mice displaying cerebral malaria (infected with *Plasmodium berghei* ANKA) as compared to those with severe but non-cerebral malaria (infected with *Plasmodium yoelii*). These miRNA are involved in the control of TGF-β and endocytosis pathways which are known to be relevant to cerebral malaria (43). These works on gene-deficient mice models need to be repeated using humanized mice models and in-vitro human infection models for those data to have any translational value. Lastly, a mutation in the miRNA−146a was reported to be linked with increased odds for *P. falciparum* infection in first-time pregnant women, thus providing an indirect evidence of miRNA-146a protective role against P. falciparum infection (44).

Although no host lncRNA has been associated with malaria etiology to date, however, high throughput analysis of Plasmodium falciparum transcriptome have uncovered several lncRNAs including lncRNA-TARE (45, 46) and lncRNA var-AS (47, 48) that play important role in the development and virulence of the parasite. Research is warranted to investigate the role of these lncRNAs in the parasite immune evasion, host cell invasion and development within the human host.

### Non-Coding RNAs in the Diagnosis of Malaria

The patients infected with *P. falciparum* manifest malaria of differing severities and clinical outcomes, such as uncomplicated malaria (UM), severe malaria, and cerebral malaria (CM). To date, few research have focused on investigating ncRNAs as biomarkers for the diagnosis of malaria. It was found that the plasma level of miR-451 and miR-16 were significantly lower in malaria patients compared to uninfected individuals, thus suggesting that plasma miR-451 and miR-16 are potentially relevant biomarkers for malaria infection (49).

Many miRNAs including miR-3135b, miR-6780b-5p, miR-1246, miR-6126, miR-3613-5p, miR-4532 and miR-6068 are upregulated in humans during the blood phase of P. falciparum infection as compared to negative controls. This upregulation was as the result of activation of host innate immunity (50). These miRNAs could be further investigated as potential blood biomarkers of the immunopathological state, thus helping in the early diagnosis of the disease. To date no lncRNA has been investigated as potential biomarker for the diagnosis of malaria.

### ncRNAs as Therapeutic Biomarkers of Malaria

MiRNA-146a is involved in innate immune response through a negative feedback loop comprising two key molecules downstream of the TLR machinery: the kinase associated with the interleukin -1 receptor (IRAK) -1 and the factor associated with the receptor of TNF (TRAF) -6. Recent studies have shown the potential of using miRNA-146a as a biopharmaceutical agent; The results of a current study suggest that miRNA-146a is involved in innate immunity against malaria, demonstrating its potency as a biopharmaceutical target (44).

## HIV/AIDS

HIV remains a major global public health issue despite the increasing access to effective HIV prevention, diagnosis, treatment, and care, including for opportunistic infections. Approximately 38.0 million people were living with HIV at the end of 2019^5^. The Human Immunodeficiency Virus (HIV) targets the immune system and weakens people’s defense against many infections and some types of cancer. As the virus destroys and impairs the function of immune cells, immunodeficiency gradually sets in the infected individual. The most advanced stage of HIV infection is Acquired Immunodeficiency Syndrome (AIDS), which can take many years to develop if not treated, depending on the individual. With the introduction of Highly Active Antiretroviral Therapy (HAART), HIV infection has become a manageable chronic health condition. There is still no cure or vaccine against HIV infection, which has been known for about forty years to date and for which research is restless (51). Many studies reported ncRNAs as novel targets for new drugs (**Table 2**). These ncRNAs influence the replication cycle of the virus.

**Table 2:** Non-coding RNAs in the etiology and control of HIV/AIDS.

Role in HIV Infection	Non-coding RNA	Action	Reference
Etiology	miR-873	Activation of HIV transcription	(52, 53)
	MALAT1		(53, 54)
	HEAL		(53, 55)
	LINC01426		(53, 56)
	LINC00173		(57)
	NEAT1	Inhibition of HIV transcription	(53, 58, 59)
	NRON		(60, 61)
	GAS5		(52)
	lincRNA-p21		(62, 63)
Diagnosis	miR-146b-5p	Downregulation in B and T-lymphocytes	(64, 65)
	miR-223		
	miR-150		
	miR-16		
	miR-191		
	lncRNA NEAT1	Presence in the plasma	
Therapeutic targets	MALAT1	Promotion of HIV transcription,	(63)
	HEAL	Action in HIV-1 latency	(53)
	SAF	Resistance of HIV-1–infected macrophages to activation of apoptotic caspases	(66)
	lincRNA-p21	HIV-1–infected macrophages	(62)
	NEAT1	Dissemination of HIV-1	(58, 59)
	GAS5	Suppression of miR-873	(52)
	NRON	HIV-1 latency	(60, 61)
	LINC00173	Regulation of cytokine levels in T cells	(57)

### Non-Coding RNAs in the Etiology of HIV Infection

**Figure 3:**
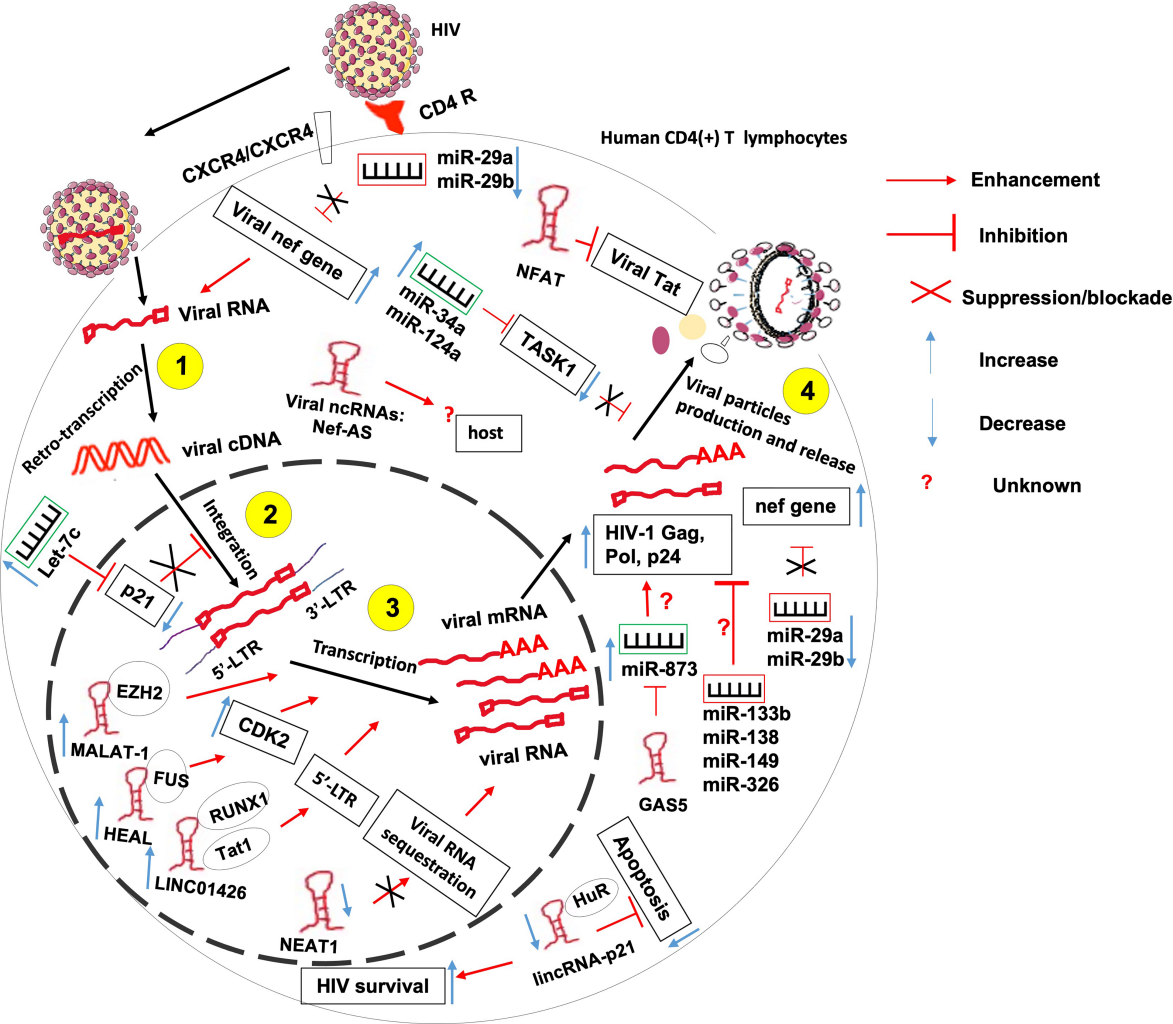
The role of non-coding RNAs in the etiology of HIV/AIDS. ncRNAs control several steps of HIV life cycle including viral RNA retro-transcription, cDNA integration, transcription and viral particles production. More research needed to identify 1- host ncRNAs that regulate viral particles release from the host and 2- the role of HIV-derived ncRNAs on the outcome of HIV-T lymphocyte interaction.

It is known that HIV interacts with the host in order to complete its replication cycle, escape immune response and persist within infected hosts. Such interactions involve both host ncRNAs and HIV-produced ncRNAs amongst other factors (**Figure 3**).

HIV hijacks host ncRNAs to promote its replication within the host. Indeed, it was observed that at the peak of HIV-1 replication, the virus downregulates the expression of miR-29a and miR-29b in CD4(+) CD8 (–) PBMCs (67). These two miRNAs were previously reported to inhibit viral replication through direct targeting of a conserved site within the viral *nef* gene (68, 69). Also, miR-873 was shown to promote HIV-1 replication in an in-vitro infection model of Jurkat and 293T cells. miR-873 promotes the production of HIV-1 gag, pol and p24 proteins through yet undefined mechanisms (52). HIV also upregulates the expression of host miRNAs let-7c, miR-34a, and miR-124a to promote its propagation. Let-7c post-transcriptionally inhibits the expression of p21 which is a known negative regulator of viral integration and RNA expression within the infected host cell (70). miR-34a and miR-124a decrease the mRNA level of TASK1 (70), which is a mammalian potassium channel known to counteract the viral Vpu -induced release of HIV virions (71).

Besides miRNAs, HIV also hijacks numbers of lncRNAs to its advantage, including Metastasis-Associated Lung Adenocarcinoma Transcript 1 (MALAT1), HIV-1 enhanced lncRNA (HEAL), LINC01426, lincRNA-p21 and Nuclear-Enriched Abundant Target 1 (NEAT1).

Indeed, induction of MALAT1 expression in T lymphocytes contributes to initial viral replication and to disease progression. Mechanistically, MALAT1 sequesters EZH2, the Histone H3K27 trimethylase of the polycomb repressive complex 2, hence releasing the epigenetic inhibition of the HIV-1 LTR promoter responsible for the latency (54).

HIV-1 infection-induced upregulation of lncRNA HEAL promotes the transcription of HIV-1 in both Monocytes-derived macrophages and in primary CD4+ T cells. Indeed, acting as a scaffold, HEAL recruits fused in sarcoma (FUS) RNA-binding protein to the promoter of CDK2 and HIV-1 LTR Which are known as activators of multiple proteins essential for HIV-1 transcription (55).

HIV1 infection-induced LINC01426 enhances HIV-1 replication thanks to its interaction with both the host RUNX1a transcription factor and viral Tat1 factors that mediate the lncRNA binding to the 5′ LTR of HIV-1 (56).

HIV-1 induces the complexation of the apoptosis-promoting lincRNA-p21 with the host protein human antigen R (HuR) and its subsequent degradation. This will lead to apoptosis inhibition and enhanced HIV survival within infected macrophages but not lymphocytes (62). It was observed that HIV-1 infection downregulates the expression of NEAT1 lncRNA leading to the reduction of the number of host-protective paraspeckle bodies, hence increased HIV-1 expression within CD4+ T lymphocytes (58). NEAT1 is also downregulated during viral reactivation from a resting state in CD4(+) T cells through an unknown mechanism leading to the promotion of HIV-1 transcription, and potentially HIV-1 dissemination (58, 59).

Contrary to the above-mentioned ncRNAs, some host ncRNAs were reported to repress the replication of HIV. These include miR-29a, miR-133b, miR-138, miR-149 and miR-326, NEAT1, noncoding repressor of Nuclear Factor of Activated T cells (NFAT or NRON), growth arrest-specific transcript 5 (GAS5), lincRNA-p21, 7SK and NEAT1.

Indeed, miR-29a is highly induced in HIV-1-infected Jurkat cells where it represses HIV replication through direct targeting of the HIV-1 nef 3’ UTR region. However, the expression of this miR-29a is significantly downregulated at the peak of HIV-1 replication as already mentioned, thus highlighting its host-protective effect against HIV-1 infection (68, 69). In addition to miR-29a, it was found that overexpression of in-silico predicted miR-133b, miR-138, miR-149 and miR-326 decrease HIV replication in various primary T cells and T cell lines. It was further shown that miR-326 acts by direct targeting of a sequence within HIV-1 (72).

As mentioned above, HIV-1 infection downregulates the expression of NEAT1 which would otherwise restrict HIV-1 replication through sequestration of unspliced viral RNA into paraspeckles (58).

The Noncoding Repressor of Nuclear Factor of Activated T cells (NFAT, or NRON) was shown to inhibit HIV-1 transcription and induce HIV-1 latency *via* induction of a proteasome-mediated HIV-1 Tat degradation, and in an NFAT-independent manner (60). This findings corroborate the previous observation that NRON is highly expressed in resting CD4(+) T lymphocytes and HIV-proteins Nef and Vpu downregulate NRON expression, hence increase HIV-1 transcription *via* mechanisms that involved NFAT transcription factor but are still not fully understood (61).

The lncRNA Growth Arrest-Specific Transcript 5 (GAS5) inhibits HIV-1 replication by acting as a competing endogenous RNA (ceRNA), suppressing the effects of the host-detrimental miR-873 (52).

It is now recognized that there are ncRNAs originating from virus genomes. For instance, the antisense transcript originating from the *Nef* region in the HIV-1 genome (73, 74) which plays important role in the transcriptional control of HIV-1, notably *via* epigenetics mechanisms (75). There is currently no studies investigating the role of HIV-1-originating ncRNAs in the regulation of the outcome of host-virus interaction.

### Non-Coding RNAs in the Diagnosis of HIV Infection

miR-146b-5p, miR-223, miR-150, miR-16, and miR-191 were found to be down regulated during HIV infection and plentifully expressed in B and T-lymphocytes, confirming a positive disease status (64, 65). Furthermore, some authors suggested that the presence of NEAT1 in plasma is a potential biomarker of HIV-1 infection (76).

### Non-Coding RNAs as Therapeutic Targets of HIV Infection

Some ncRNAs have been associated to the treatment of HIV infection. miR-29a can be used as an indicator for the on-treatment disease evolution CD4 count or zenith HIV viral load (77). Its expression is associated to markers of HIV infection in long-term survivors, treatment-experienced patients (77). 7SK, by its pseudouridylation, can inhibit HIV transcription and escape from latency, suggesting it may be a new target for eliminating latent viral reservoirs (63, 78).

Many lncRNAs can be related to therapeutic research of HIV infection. MALAT1, as a promoter of HIV transcription, is a potential therapeutic target (54, 63). uc002yug.2, by activating latent HIV and HIV replication, can be a potential therapeutic target (56, 63). HEAL may be an attractive therapeutic target to inhibit HIV-1 latency, particularly considering that it is only upregulated in infected CD4+ and macrophages (53, 55, 63). SAF, involved in the resistance of HIV-1–infected macrophages to activation of apoptotic caspases, is a potential therapeutic target specifically intended for HIV cellular reservoirs (63, 66). lincRNA-p21 can constitute a novel therapeutic intervention strategy for HIV infection in macrophages (62, 63). NEAT1 is a feasible target for HIV treatment that involves the reactivation of latent HIV (58, 59, 63). GAS5, by suppressing miR-873, may be a novel biomarker for antiviral drugs and potential target for HIV treatment (52, 63). NRON, as an actor of HIV-1 latency, may be a novel target for reversing viral latency (60, 61, 63). TAR-gag, as an “RNA machine” of HIV genetic regulation, is a novel therapeutic target to reverse viral latency (63, 79). HIV-encoded lncRNA, as an epigenetic brake to regulate viral transcription, is a novel therapeutic target to inhibit the emergence of viral latency (51, 63, 75). LINC00173, which regulates cytokine levels in T cells, is a new therapeutic target for immunotherapy (57, 63).

## Leishmaniasis

Leishmaniasis is a neglected tropical disease caused by infection with *Leishmania* parasites, which are spread by the bite of phlebotomine sand flies. There are different forms of leishmaniasis in people and the most common are Cutaneous Leishmaniasis (CL), which causes skin sores, and Visceral Leishmaniasis (VL), which affects several internal organs (generally spleen, liver, and bone marrow)^6^.

Leishmaniasis is prevalent on every continent except Australia and Antarctica. It is difficult to estimate the number of new cases that may vary over time. For CL, estimates of the number of new cases per year have ranged from approximately 700,000 to 1.2 million or more. For VL, the estimated number of new cases per year may have decreased to <100,000, but previous estimates ranged up to 400,000 or more cases^7^. If not treated, severe cases of visceral leishmaniasis typically are fatal.

Before considering treatment of leishmaniasis, it is essential to make sure the diagnosis is correct. It can be done by detecting Leishmania parasites (or DNA) in tissue specimens from skin lesions (CL) or bone marrow (VL). This tissue sampling is an invasive method. Conversely, the diagnosis of disease with the help of a biomarker is a non-invasive tool that has shown to have an important function in early diagnosis of infection. There is no cure for leishmaniasis and chemotherapy is threatened by limited efficacy coupled with the development of resistance and other side effects (80). Leishmania parasites elude the host defensive. Some ncRNAs have been reported as biomarkers for the diagnosis and the treatment of leishmaniasis (**Table 3**).

**Table 3:** Non-coding RNAs in the etiology and control of leishmaniasis.

Role in Leishmaniasis	Non-coding RNA	Action	Reference
Etiology	miR-210	Downregulation of NF-κB mediated pro-inflammatory immune responses	(81)
Therapeutic targets	miR-361-3p	Its high expression in cutaneous leishmaniasis lesions	(82)
	miR-193b	Influence in the expression of genes related to the inflammatory response observed in localized cutaneous leishmaniasis	(83)
	miR-671		
Diagnosis	lncRNA 7SL	It makes the difference between *Leishmania* species infections	
Vaccine development	miR-135	Biasing the Th2 response toward protective Th1 type	(84, 85)
	miR-126		

### Non-Coding RNAs in the Etiology of Leishmaniasis

**Figure 4:**
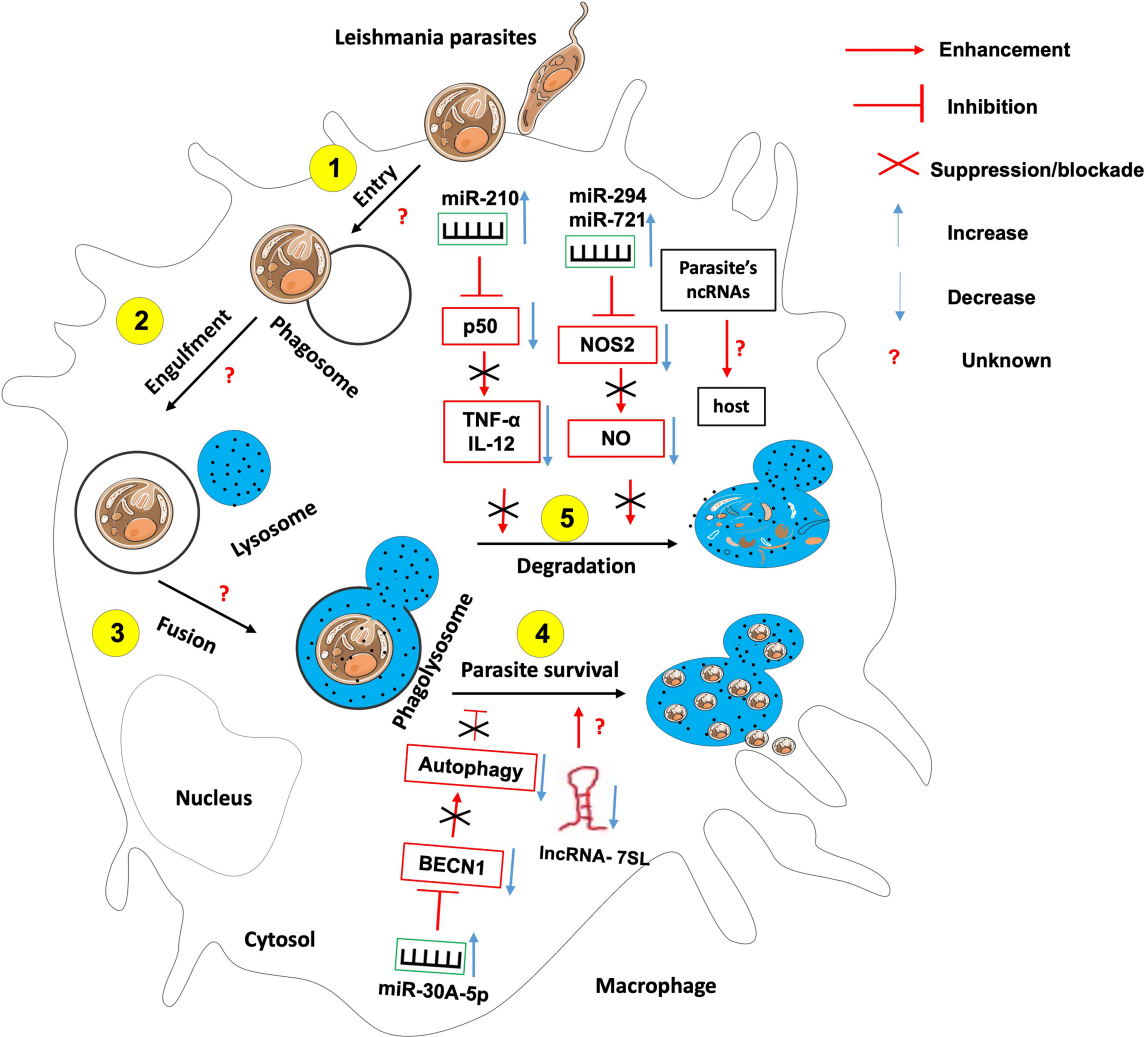
The role of non-coding RNAs in the etiology of leishmaniasis. Most studies have identified ncRNAs that enhance host effector killing functions against leishmania parasites as well as those that favor parasite survival and persistence within the infected host. There is knowledge gap about 1- host ncRNAs that regulate the parasite entry within the host, its engulfment within phagosome and the fusion between phagosome and lysosomes; 2- the role of Leishmania-derived ncRNAs on the outcome of leishmania-macrophage interaction.

Like many sophisticated intracellular pathogens, Leishmania has evolved mechanisms to modify the host responses to ensure their intracellular differentiation and multiplication. The parasite does so through the manipulation of the host factors including several miRNAs (**Figure 4**).

For instance, Leishmania *donovani* infection creates hypoxic conditions leading to HIF-1a-mediated induction of miR-210 in infected macrophages (81, 86). This miR-210 was shown to promote the survival of the parasite within the host by targeting the NFkb subunit p50 and subsequently downregulating the expression of pro-inflammatory cytokines TNF-α and IL-12 while upregulating the anti-inflammatory cytokine IL-10 within the infected macrophage (81). Also, L. donovani interferes with host autophagy by inducing the expression of host miR-30A-5P which in turn will downregulate the expression of the pro-autophagic BECN1 protein, hence the increased survival of the parasite within the infected macrophage (87). *Leishmania amazonensis* promotes its survival within the infected host by upregulating miR-294 and miR-721. These miRNAs target the nitric oxide synthase 2 (NOS2) mRNA at the post-transcriptional level, then leading to decreased nitric oxide production and increased arginase activity within the infected macrophage (88). miR-21 and miR-146b-5p are significantly downregulated *in monocytes-derived dendritic cells following L. donovani and L. major infection. In-silico prediction have identified* SMAD7 and TRAF6, two members of the TGF-β signaling pathway as targets for these two miRNAs (89). It was found that *L. major* infection led to the down-regulation of miR-10a in Foxp3^+^ Treg cells. This miR-10a decreased IFNγ and enhanced the suppressive function of Treg cells (90).

*Leishmania* promastigotes and amastigotes infection represses the expression of the lncRNA 7SL RNA, an important component of the signal recognition particle in macrophages. This will convert these cells into permissible hosts favorable to the establishment and hiding of the parasite within the macrophages’ phagolysosomes (91).

It is still unknown whether and which host ncRNAs control leishmania parasites entry, engulfment within phagosomes and fusion of phagosomes with lysosomes.

An investigation on Leishmania major genome revealed that there are 1884 uniquely expressed ncRNAs in that parasite, some of which were recently shown to possess protein coding potential (92). The contribution of these parasite-derived ncRNA to the outcome of parasite-host interaction still need investigation.

### Non-Coding RNAs in the Diagnosis of Leishmaniasis

The 7SL RNA gene can be used for diagnosis of human leishmaniasis (93). Diagnosis with the help of 7SL RNA is rapid, sensitive, specific, and simple (93).

### Non-Coding RNAs in the Treatment of Leishmaniasis

Some authors suggested that miR-361-3p is a prognostic biomarker in cutaneous leishmaniasis lesions caused by *Leishmania braziliensis* (82). miR-193b and miR-671 were associated with a good response to treatment of Human localized cutaneous leishmaniasis caused by *Leishmania braziliensis* (83). Targeting of let-7a with Locked Nucleic Acid (LNA) Antisense Oligonucleotides (ASOs) was shown to increase L. major-infected MDMs apoptosis and necrosis, therefore, targeting let-7a was suggested as a potential therapeutic approach (94).

### Non-Coding RNAs in Vaccine Development Against Leishmaniasis

miRNA21 has been recently shown to negatively associate with IL-12 production and priming of protective Th1 response, suggesting declining levels of miRNA-21 as a potential biomarker of safety and immunogenicity in anti-leishmanial vaccines (84, 95).

Therapeutic vaccines may be developed by targeting miRNA-135 and−126 that bias the Th2 response toward protective Th1 type (84, 85).

## African Trypanosomiasis

Also known as sleeping sickness, Human African Trypanosomiasis (HAT) is a neglected tropical disease caused by microscopic parasites of the species *Trypanosoma brucei* whose vector is an insect of the genus Glossina: the tsetse fly^8^. Two subspecies of *Trypanosoma brucei* are responsible of human disease: *T. b. gambiense* in 24 countries in west and central Africa, and *T. b. rhodesiense* in 13 countries in eastern and southern Africa^9^.

HAT is curable with medication but is fatal if left untreated. Diagnosis must be made as early as possible to avoid progressing to the neurological stage in order to prevent complicated and risky treatment procedures (95). Diagnosis is made clinically or by light microscopy; which are both insensitive and require certain skills^10^. The use of biomarkers such as ncRNAs could enhance sensitivity.

### Non-Coding RNAs in the Etiology of African Trypanosomiasis

The alteration of nine miRNAs Including miR-193b, miR-338 (upregulated), miR-199a-3p, miR-27b and miR-126* (downregulated) has been identified in the peripheral blood of HAT patients (96). They were non-specific and some of them were previously reported changed during other infectious diseases or cancer. They might be a mirror lymphocyte activation or inflammation observed in HAT (96). However, the mechanisms of action of these microRNAs are still to be investigated.

### ncRNAs as Diagnostic Biomarkers of African Trypanosomiasis

The SL-RNA was described as an attractive molecular target of the sleeping sickness (97). Later, the small RNA derived from the non-coding 7SL RNA was detected at high levels in the serum of infected cattle (98). This ncRNA is highly sensitive and can be detected before the onset of parasitemia as well as during periods where there is subpatent parasitemia by microscopy (98). It can also make the difference between infections with *Trypanosoma brucei*, *Trypanosoma congolense* and *Trypanosoma vivax*; providing the basis for the development of a cheap, non-invasive and highly effective diagnostic test for trypanosomiasis (98).

### ncRNAs as Therapeutic Biomarkers of African Trypanosomiasis

The therapy of trypanosomiasis is currently based on anti-trypanosome drugs. No therapeutic field with ncRNAs has yet been investigated. Research should investigate this domain and see if ncRNAs might be useful for monitoring the treatment of this disease.

## Leprosy

Also called Hansen’s disease, leprosy is a chronic infectious disease caused by *Mycobacterium leprae*. This slow-growing, obligate intracellular bacterium is the only known bacterium that infects Schwann cells of peripheral nerves. In addition, M. leprae infects macrophages and dendritic cells (99). M leprae mainly affects the skin, the peripheral nerves, mucosa of the upper respiratory tract, and the eyes^11^ ^12^. According to official figures from 159 countries from the 6 WHO Regions, 208 619 new leprosy cases were globally registered in 2018^13^.

Leprosy presents in many clinical forms with the two extremes being the Tuberculoid and the Lepromatous forms. In the tuberculoid forms (TT and BT), bacilli are absent or rarely found in neural branches, macrophages, or mononuclear cells of the papillary dermis. the disease is self-limited. On the other hand, in lepromatous forms (BL and LL), the bacilli are abundant and can parasitize practically all tissues, hence the disease is disseminated. These clinical forms can be recognized with the naked eye. To confirm the diagnosis, a sample of skin or nerve can be examined under the microscope and tests may also be done to differentiate it from other skin diseases^14^. These sampling techniques are invasive. Alternatively, to diagnose an infection early, biomarkers are useful and non-invasive. Some ncRNAs have been reported as biomarkers for the diagnosis of leprosy (**Table 4**).

**Table 4:** Non-coding RNAs in the etiology and control of leprosy.

Role in leprosy	Non-coding RNA	Action	Reference
Etiology	miR-181a	Rheostat of intrinsic antigen sensitivity during LT development	(100)
	miR-146a	Reduction of the TNF expression	(101)
	miR-21	Downregulation of host defense genes to evade vitamin D antimicrobial pathway	(102)
Diagnosis	miR-101	Modulation of the host immune response in leprosy	(103)
	miR-196b		
	miR-27b		
	miR-29c		

### Non-Coding RNAs in the Etiology and Occurrence of Leprosy

**Figure 5:**
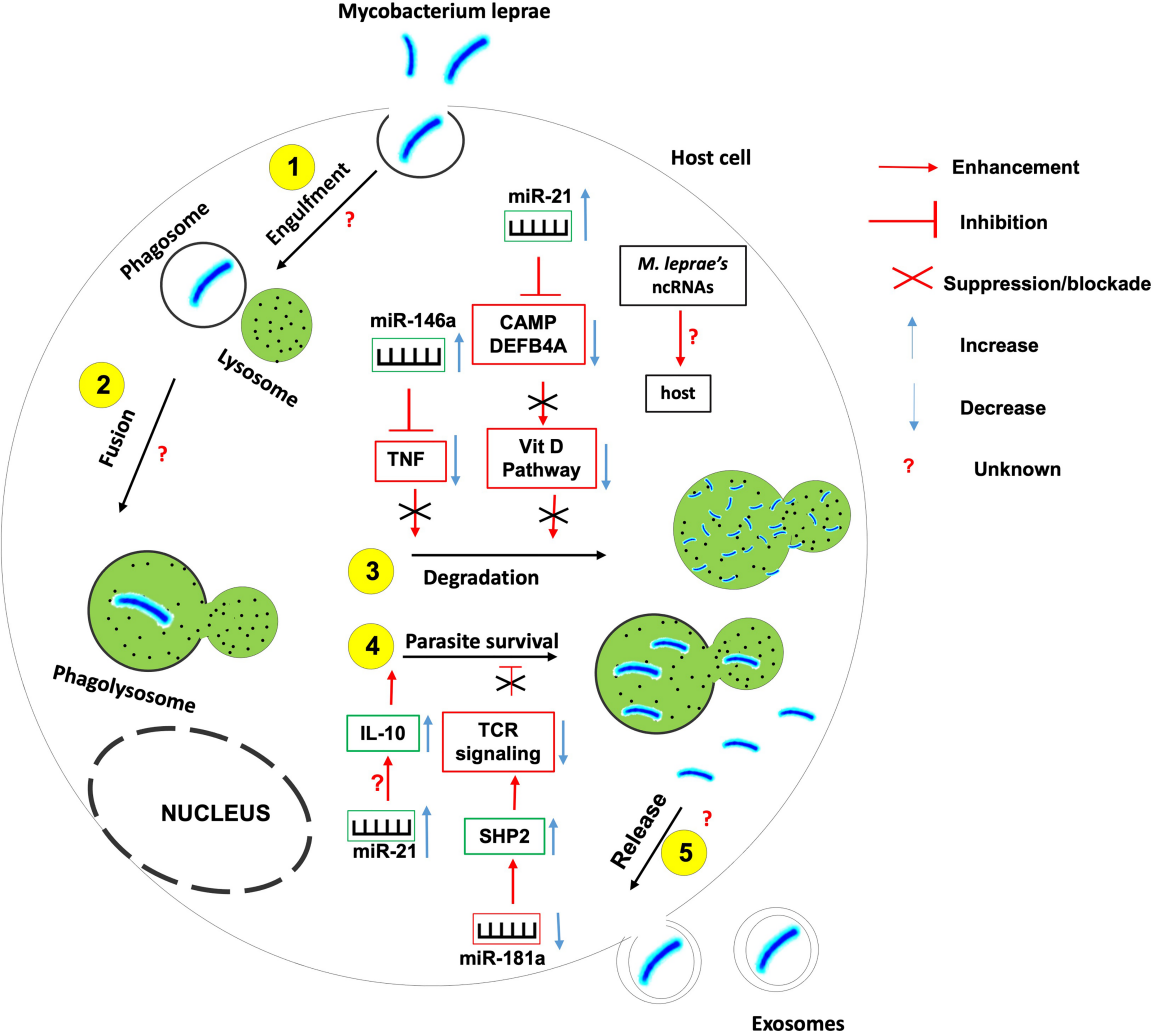
The role of non-coding RNAs in the etiology of leprosy. Several ncRNAs are found to be instrumental in the induction or inhibition of the host effector killing functions against Mycobacterium leprae. More research needed to identify 1- host ncRNAs that regulate the parasite entry within the host, its engulfment within phagosome and the fusion between phagosome and lysosomes; 2- the role of M. leprae-derived ncRNAs on the outcome of mycobacterium-macrophage interaction.

It is now recognized that ncRNAs play important roles in the deregulation of the immune response in the varied and polymorphic cells targeted in the leprosy skin lesions onset (99, 104, 105). Specific miRNAs regulated during infection either stimulate the immune response or facilitate immune evasion by pathogens (**Figure 5**).

For instance, miR-21 is upregulated in *M. leprae*- infected monocytes in which it downregulates the expression of genes encoding 2 vitamin D-dependent antimicrobial peptides CAMP and DEFB4A, hence used by the mycobacterium to evade the vitamin D-antimicrobial pathway (102). Also, miR-21 involves in the indirect upregulation of IL-10 and is differentially expressed in humans with progressive lepromatosis.

miR-146a expression is upregulated in hosts infected with live M. leprae. miR-146a bears a single nucleotide polymorphism associated with the risk of developing leprosy, hence the expression of this miR is dependent on the host genotype (101). Carriers of the miR-146a C allele have been shown to express high levels of mature miR-146a coupled to a reduced expression of TNF (Tumor Necrosis Factor) with a susceptibility to leprosy; suggesting that miR-146a negatively influences the secretion of TNF by controlling its level of expression.

miR-181a expression is downregulated in *M* leprae-infected T lymphocytes. This downregulation correlates with the increased expression of miR-181a target SHP2, a phosphatase involved in the inhibition of T cell receptor signaling (106). Indeed, higher miR-181a expression correlates with greater T cell sensitivity in immature T cells (107) suggesting that the downregulation of miR-181a expression in *M.* leprae-infected T lymphocytes involves in the prevailing T cell hyporesponsiveness during leprosy progression.

Recent studies have identified differentially expressed piwiRNAs (piRNAs) in leprosy skin lesions from tuberculoid tissue, lepromatous tissue, and healthy subject tissues (108). This class of small ncRNAs is closely related to miRNAs and its study will provide additional clues on the contribution of ncRNAs to the onset, development, and progression of leprosy. The contribution of M. leprae-derived ncRNAs also need to be explored.

### ncRNAs as Diagnostic Biomarkers of Leprosy

Early diagnosis of leprosy is very important to control the disease and put in place preventive measures. Currently, the diagnosis of leprosy is based on clinical examination and skin biopsy. Techniques based on PCR and serological analysis have been developed but have not made it possible to diagnose leprosy with acceptable specificity and sensitivity given the different clinical forms and/or the bacterial load. However, the identification of biomarkers allows the diagnosis of leprosy with greater sensitivity and specificity. Due to the influence of the host’s genetic makeup on the development of leprosy and the genetic variants associated with it. The expression profile of ncRNAs and more precisely miRNAs is a key element exploited in the development of reliable diagnostic and prognostic biomarkers. miR-101, miR-196b, miR-27b and miR-29c have been differentially expressed in different cell types: macrophages, LT, epithelial cells, dendritic cells, mast cells with establishment of an immune/inflammatory microenvironment in *M. leprea* infection. These miRNAs are linked to immune genetic targets and could modulate the host immune response in leprosy influencing its outcome. Thus, miR-101, miR-196b, miR-27b and miR-29c were identified as good discriminators in the polar forms of leprosy (LL: lepromatous leprosy and TT: tuberculoid tuberculoids) and between physiological state and pathological state (103) with high levels of sensitivity and specificity.

### ncRNAs as Therapeutic Biomarkers of Leprosy

Although drug treatment has been successful, leprosy still affects people all over the world. The treatment of leprosy is based mainly on polychemotherapy, which has so far remained the only strategy for the treatment and elimination of leprosy (109). hsa-miR-142-3p, hsa-miR-142-5p, hsa-miR-146b-5p, hsa-miR-342-3p, hsa-miR-361-3p, hsa-miR-3653 hsa-miR-484 and hsa-miR-1290 were reported deregulated in leprosy and could serve as therapeutic markers (7, 110).

### Non-Coding RNAs in Vaccine Development Against Leprosy

No ncRNA-based vaccine has been developed so far against leprosy. Researchers can explore this new research avenue.

## Conclusion

There is a growing interest in the role of host miRNAs and lncRNAs in the etiology of major human tropical diseases and the prospect of targeting these ncRNAs species as biomarkers for the early diagnosis, treatment response and vaccine development against these diseases. Although most research have shed light on the involvement of these ncRNAs in the onset and development of TB, HIV/AIDS and Malaria, but few have yet attempted to assess the potential of these ncRNAs as diagnosis biomarkers, adjunctive therapeutic targets and vaccine candidates. There is also a lack of information about the contribution of pathogen-released ncRNAs to host immune evasion and disease onset. There is a knowledge gap on the role of host miRNAs and lncRNAs in the etiology, diagnosis and vaccine development against neglected human tropical diseases. Especially, more research is warranted to understand the role of these ncRNAs in the etiology of African trypanosomiasis and the assessment of the diagnostic potential of ncRNAs for African trypanosomiasis. The potential targeting of ncRNAs for adjunctive therapy and vaccine development against leishmaniasis, African trypanosomiasis and leprosy are also new avenues to explore.

## Author Contributions

OT designed the writing plan and drew all the figures. CM and JAN did literature search on Leishmaniasis and HIV/AIDS. CK and USFS did literature search on Tuberculosis and Malaria. NN and FJT did literature search on Leprosy and Trypanosomiasis. OT, CM, CK, and NN wrote the draft. AM and AN proofread the manuscript. All authors contributed to the article and approved the submitted version.

## Conflict of Interest

The authors declare that the research was conducted in the absence of any commercial or financial relationships that could be construed as a potential conflict of interest.

## Publisher’s Note

All claims expressed in this article are solely those of the authors and do not necessarily represent those of their affiliated organizations, or those of the publisher, the editors and the reviewers. Any product that may be evaluated in this article, or claim that may be made by its manufacturer, is not guaranteed or endorsed by the publisher.

## Footnotes

1
https://www.cdc.gov/malaria/about/biology/index.html

2
https://www.who.int/news-room/fact-sheets/detail/malaria

3
https://www.who.int/news-room/fact-sheets/detail/malaria

4
https://www.cdc.gov/malaria/about/faqs.html

5
https://www.who.int/news-room/fact-sheets/detail/hiv-aids

6
https://www.cdc.gov/parasites/leishmaniasis/index.html

7
https://www.cdc.gov/parasites/leishmaniasis/epi.html

8
https://www.cdc.gov/parasites/sleepingsickness/index.html

9
https://www.who.int/news-room/fact-sheets/detail/trypanosomiasis-human-african-(sleeping-sickness

10
https://www.cdc.gov/parasites/sleepingsickness/diagnosis.html

11
https://www.cdc.gov/leprosy/index.html

12
https://www.who.int/news-room/fact-sheets/detail/leprosy

13
https://www.who.int/news-room/fact-sheets/detail/leprosy

14
https://www.cdc.gov/leprosy/index.html
